# Treatment with a New Barbituric Acid Derivative Exerts Antiproliferative and Antimigratory Effects against Sorafenib Resistance in Hepatocellular Carcinoma

**DOI:** 10.3390/molecules25122856

**Published:** 2020-06-20

**Authors:** Yi-Jen Liao, Shih-Ming Hsu, Chia-Ying Chien, Yuan-Hsi Wang, Ming-Hua Hsu, Fat-Moon Suk

**Affiliations:** 1 School of Medical Laboratory Science and Biotechnology, College of Medical Science and Technology, Taipei Medical University, Taipei 110, Taiwan; yjliao@tmu.edu.tw (Y.-J.L.); m609108003@tmu.edu.tw (C.-Y.C.); m609105001@tmu.edu.tw (Y.-H.W.); 2 Department of Biomedical Imaging and Radiological Sciences, National Yang-Ming University, Taipei 11221, Taiwan; smhsu@ym.edu.tw; 3 Department of Chemistry, National Changhua University of Education, Changhua 50007, Taiwan; 4 Division of Gastroenterology, Department of Internal Medicine, Wan Fang Hospital, Taipei Medical University, Taipei 116, Taiwan; 5 Department of Internal Medicine, School of Medicine, College of Medicine, Taipei Medical University, Taipei 110, Taiwan

**Keywords:** barbituric acid derivatives, drug resistance, liver cancer

## Abstract

Hepatocellular carcinoma (HCC) is a common cause of cancer death worldwide. Sorafenib, a multikinase inhibitor, is the first-line drug approved by the Food and Drug Administration (FDA) for the treatment of patients with advanced HCC. However, most patients who continuously receive sorafenib may acquire resistance to this drug. Therefore, it is important to develop a new compound to treat liver cancer and sorafenib-resistant liver cancer. Barbituric acid derivatives have been used as antiasthmatic drugs in the clinic. We previously reported that a novel barbituric acid derivative inhibited carbon tetrachloride-induced liver fibrosis in mice, but its effects on liver cancer remain unknown. Thus, the purpose of this study was to investigate the antitumor effect of barbituric acid derivatives on HCC cells and sorafenib-resistant HCC cells (HCC-SRs). Our findings reveal that one of the barbituric acid derivatives, BA-5, significantly inhibited HCC and HCC-SR cell viability in a dose- and time-dependent manner. Therefore, compound BA-5 was selected for further experiments. Western blot data revealed that BA-5 treatment decreased the phosphorylation of AKT/p70s6k without affecting the MAPK pathway and increased cleaved PARP and cleaved caspase-7 in both HCC and HCC-SR cells. Since epithelial-mesenchymal transition plays a significant role in regulating cancer invasion and migration, we used the wound healing assay to evaluate the antimigratory effect of compound BA-5. The results showed that BA-5 treatment inhibited HCC and HCC-SR cell migration and reduced Vimentin protein expression. These results were confirmed by microarray analysis showing that BA-5 treatment influenced cancer cell motility and growth-related pathways. In the xenograft mouse model experiment, BA-5 administration significantly inhibited HCC cancer cell growth in mice. Furthermore, the combination of BA-5 with a low dose of regorafenib synergistically inhibited HCC-SR cell proliferation. In conclusion, our study showed that the barbituric acid derivative BA-5 is a new candidate for HCC and sorafenib-resistant HCC therapy.

## 1. Introduction

Liver cancer is the leading cause of cancer-associated death [1]. Liver cancer consists of a diverse group of malignant tumors, which include hepatocellular carcinoma (HCC), intrahepatic cholangiocarcinoma, and other tumors. Among liver cancers, HCC comprises 70–80% of cases [2]. The risk factors of cirrhosis, which include viral hepatitis, such as Hepatitis B virus infection, and HCV infection, and alcohol consumption [3], can be the most common cause for developing HCC because a great number of HCCs evolve from hepatic cirrhosis [4]. In addition, other main risk factors associated with HCC patients include non-alcoholic fatty liver disease (NAFLD) and non-alcoholic steatohepatitis [5]. The development of HCC is complex and includes cancer cell proliferation and metastasis [6]. The epithelial to mesenchymal transition is a process in which epithelial cells transition to mesenchymal cells, which are involved in invasion and metastasis in HCC [7]. The curative treatment options for HCC, such as radiofrequency ablation, hepatic resection, and liver transplantation, depend on the liver function and tumor size [8]. However, most HCC patients are diagnosed with advanced stage disease and are not eligible for surgery. Sorafenib is a first-line systemic therapy approved for the treatment of advanced HCC by the U.S. Food and Drug Administration (FDA). Sorafenib is an orally administered multikinase inhibitor that decreases tumor cell proliferation by blocking Raf-1, wild-type B-Raf and the mitogen-activated protein kinase (MAPK) extracellular signaling-regulated kinase (ERK) signaling pathway. In addition, sorafenib also inhibits tumor angiogenesis by targeting many receptor tyrosine kinases, including vascular endothelial growth factor receptor (VEGFR-1), VEGFR-2, VEGFR-3, hepatocyte factor receptor (c-KIT), Fms-like tyrosine kinase (FLT-3), and platelet-derived growth factor receptor (PDGFR)-b [9]. The Sorafenib Hepatocellular Carcinoma Assessment Randomized Protocol (SHARP) trial showed that sorafenib increased the median overall survival (mOS) versus placebo (mOS: 10.7 versus 7.9 months). Sorafenib extended the overall survival to 6.5 months compared with 4.2 months with placebo in the Asia-Pacific region [10]. Recent clinical studies illustrated that sorafenib has effective antitumor activity in thyroid cancer, myeloid leukemia, mesothelioma, renal cell carcinoma, and prostate cancers [11]. Although sorafenib has many benefits, side effects associated with it have been reported. Its adverse effects include hypertension, gastrointestinal disturbances, renal failure, and hand-foot-skin reaction [12]. In addition, owing to the biological heterogeneity of HCC, some patients are primarily resistant to sorafenib. Moreover, acquired sorafenib resistance in HCC patients has been observed in the clinic [13]. The mechanisms accounting for sorafenib resistance are complex and unclear. For instance, long-term exposure to sorafenib activated the phosphatidylinositol 3-kinase (PI3K)/AKT signaling pathway and resulted in sorafenib resistance in HCC [14]. Epithelial-mesenchymal transition (EMT) is involved in sorafenib-resistant HCC cells and is mediated by activation of the PI3K/AKT pathway [15]. Due to sorafenib resistance, it is essential to develop a new drug to against HCC and sorafenib-resistant HCC.

**Figure 1:**
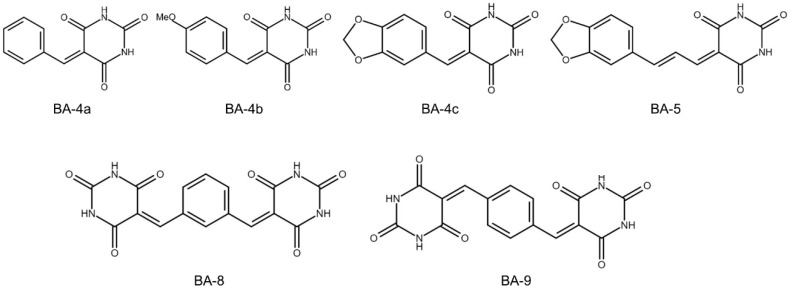
Structure of barbituric acid derivatives.

Barbituric acids are a class of aromatic hydrocarbons first synthesized by German chemist Adolph von Baeyer in 1864 [16]. The first active barbituric acid derivatives, synthesized by Fischer and von Mering in 1903, were discovered to induce sleep in humans [17]. The derivatives of barbituric acid play an important role in biology and medicine. Therefore, derivatives of barbituric acid, known as barbiturates, have appilications in central nervous system therapy, sedation, and anesthetics and have been used as anticonvulsants [18,19]. Derivatives of barbituric acid have a wide range of biological activities, including antioxidant, antiurease, and antibacterial activities [20]. In addition, 5-benzylidene barbiturate derivatives showed inhibitory effects against bacteria and mushroom tyrosinase [21]. Derivatives of barbituric acid demonstrated antibacterial, antifungal, and antidiabetic action [22]. New synthesized barbituric acid exhibited immunomodualtory and anti-HIV activities [23]. In addition, barbiturates present anticancer activity in melanoma [24,25,26]. Recent studies demonstrated that novel barbituric acid derivatives inhibited the development of NAFLD in male Wistar rats [27,28]. These findings suggest that barbituric acid derivatives are a potential therapy for liver disease. Recently, we synthesized a series of six barbituric acid derivatives (Figure 1) and found that one of the compounds exhibited the best ability to ameliorate TGF-β1-induced hepatic stellate cell activation and liver fibrosis in mice [29]. In this study, we examined the anti-liver cancer effects of these barbituric acid derivatives on liver cancer cells and sorafenib-resistant liver cancer cells and clarified the underlying mechanisms.

## 2. Results

### 2.1. BA-5 Possessed the Best Ability to Decrease Cell Viability in Both Parental and Sorafenib-Resistant HCC Cells

**Figure 2:**
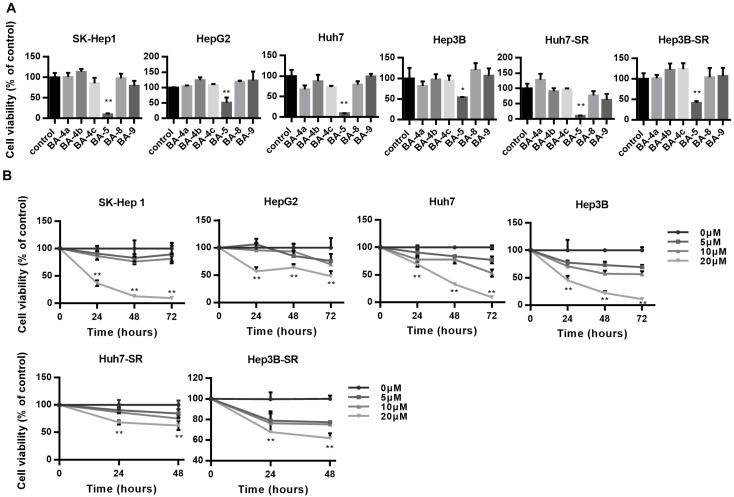
BA-5 treatment decreased HCC and HCC-SR cell viability. (**A**) SK-Hep 1, HepG2, Huh7, Hep3B (3 × 10^4^/well), Huh7-SR and Hep3B-SR (2.5 × 10^4^/well) cells were seeded in 96-well plates incubated with or without the compounds BA-4a, BA-4b, BA-4c, BA-5, BA-8, and BA-9 (20 µM) for 72 h, and cell viability was assessed using an MTT assay. (**B**) SK-Hep 1, HepG2, Huh7, Hep3B (3 × 10^4^/well) cells were seeded in 96-well plates and treated with the indicated dose of BA-5 for 24, 48, and 72 h. Huh7-SR and Hep3B-SR (2.5 × 10^4^/well) were seeded in 96-well plates and treated with the indicated dose of BA-5 for 24, 48h, and cell viability was detected by using an MTT assay. *, *p* < 0.05; **, *p* < 0.01. compared to the control group.

To determine which of the six different barbituric acid derivatives has the best antiproliferative effect, we used four different HCC cell lines, SK-Hep 1, HepG2, Huh7, and Hep3B and two sorafenib-resistant HCC cells, Huh7-SR and Hep3B-SR in the experiment. The results showed that cells treated with BA-5 for 72 h had the most significantly inhibited cell viability in all four HCC cell lines and two sorafenib-resistant HCC cells compared with that of the other candidates (Figure 2A). Next, we used different concentrations of BA-5 ranging from 0 to 20 µM and treated the four different HCC cell lines for 24, 48, and 72 h. As shown in Figure 2B, BA-5 significantly inhibited the growth of the four HCC cell lines in a dose- and time-dependent manner. Likewise, Huh7-SR and Hep3B-SR cells were treated with 0, 5, 10, 20 µM BA-5 for 24 and 48 h. Similar to the results of the parental cells, treatment with BA-5 suppressed Huh7-SR and Hep3B-SR cell proliferation in a dose- and time-dependent manner (Figure 2B). These data suggest that BA-5 has the best cytotoxic effect on both parental and sorafenib-resistant HCC cells.

### 2.2. BA-5 Treatment Inhibited HCC and HCC-SR Cell Proliferation by Blocking AKT Signaling Pathways

**Figure 3:**
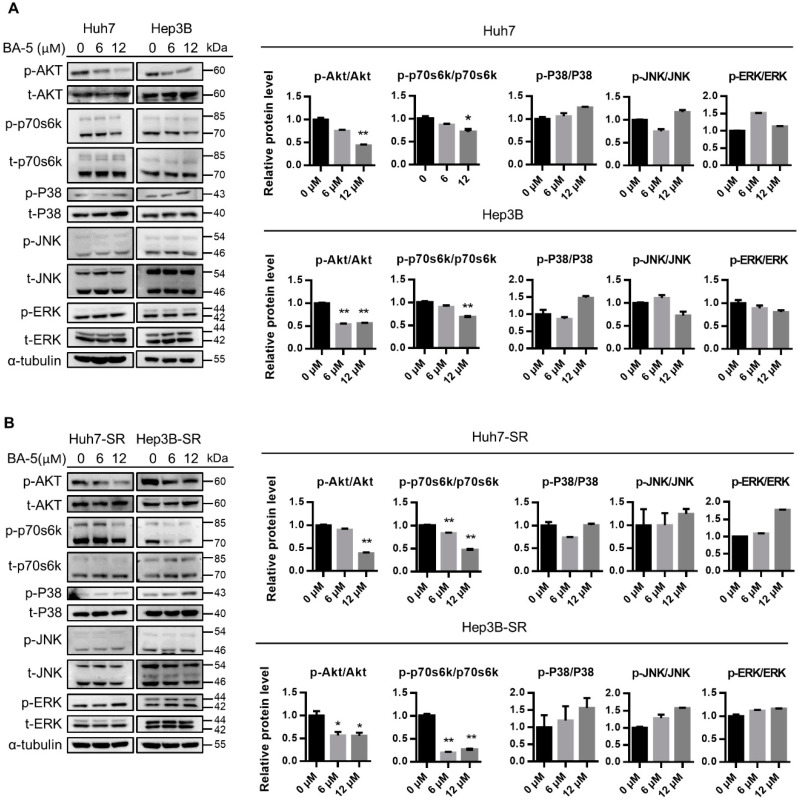
Treatment with BA-5 reduced phosphorylated AKT and phosphorylated p70s6k expression in HCC and HCC-SR cells. (**A**,**B**) Parental HCC cells (1.6 × 10^5^/well Hep3B and Huh7) and sorafenib-resistant HCC cells (1.4 × 10^5^/well Hep3B-SR and Huh7-SR) were seeded in 6-well plates and treated with 0, 6, and 12 µM BA-5 for 48 h. The protein expression levels of AKT, p70s6k, ERK, JNK, and p38 were evaluated by western blot. α-Tubulin served as a loading control. Protein quantification was performed by using Image J software. *, *p* < 0.05; **, *p* < 0.01 compared to the black bar.

To study the mechanisms underlying the antiproliferative effect of compound BA-5 in HCC and HCC-SR cells, we examined protein phosphorylation in both the AKT and MAPK signaling pathways. As shown in Figure 3A, HCC cells treated with BA-5 at concentrations of 6 and 12 µM reduced AKT and p70s6k phosphorylation. In contrast, the phosphorylation of MAPK/ERK pathway-related proteins, including ERK, JNK, and P38, was not reduced after BA-5 treatment (Figure 3A). A similar pattern was observed in HCC-SR cells. BA-5-treated Huh7-SR and Hep3B-SR cells showed reduced p-AKT and p-p70s6k expression, while p-ERK, p-JNK, and p-P38 remained unchanged (Figure 3B). These findings indicated that BA-5 inhibited HCC and HCC-SR cell proliferation by blocking the AKT/p70s6k pathway.

### 2.3. Treatment with BA-5 Activated the Apoptosis Signaling Pathway

**Figure 4:**
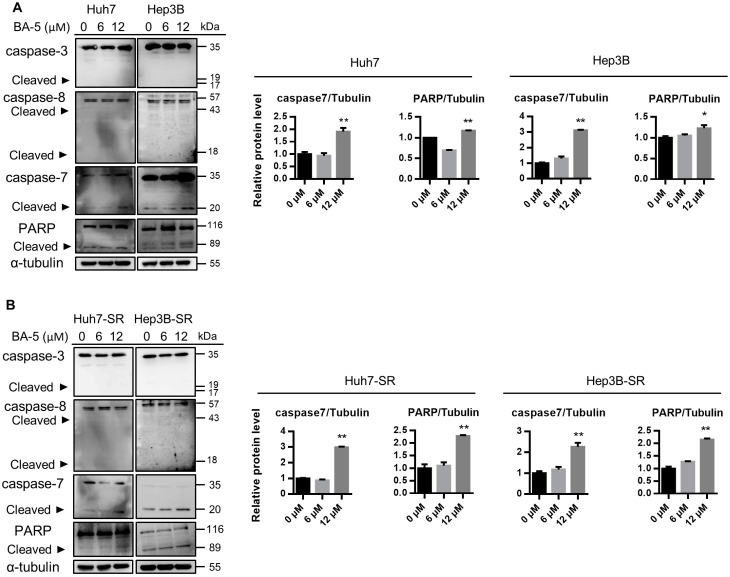
BA-5 treatment activated caspase-7 and PARP cleavage. (**A**) Parental HCC cells (1.6 × 10^5^/well Hep3B and Huh7) and (**B**) sorafenib-resistant HCC cells (1.4 × 10^5^/well Hep3B-SR and Huh7-SR) were seeded in 6-well plates and treated with 0, 6, and 12 µM BA-5 for 48 h. The protein expression levels of cleaved caspase-7 and PARP were evaluated by western blot. α-Tubulin served as a loading control. Protein quantification was performed by using Image J software. *, *p* < 0.05; **, *p* < 0.01 compared to the black bar.

Next, we investigated whether BA-5 decreases cell growth by triggering apoptotic signaling activation. The activities of caspase-8, caspase-3, caspase-7 and PARP were determined by western blot analysis. The results showed that BA-5 treatment didn’t trigger the cleavage of caspase-8 and caspase-3. On the contrary, BA-5 treatment enhanced the protein cleavage of caspase-7 and PARP in both Huh7 and Hep3B cells (Figure 4A, left). In addition, protein quantitative analyses of cleaved caspase-7 and PARP levels showed a significant increase in BA-5-treated cells compared with that of controls (Figure 4A, right). Similarly, BA-5-treated Huh7-SR and Hep3B-SR cells showed increased cleavage of caspase-7 and PARP instead of cleaved caspase-3 and cleaved caspase-8 (Figure 4B). These results indicated that BA-5 induced cell apoptosis through the activation of caspase-7/PARP-dependent signaling.

### 2.4. BA-5 Treatment Inhibited the Migration Ability of HCC and HCC-SR Cells

**Figure 5:**
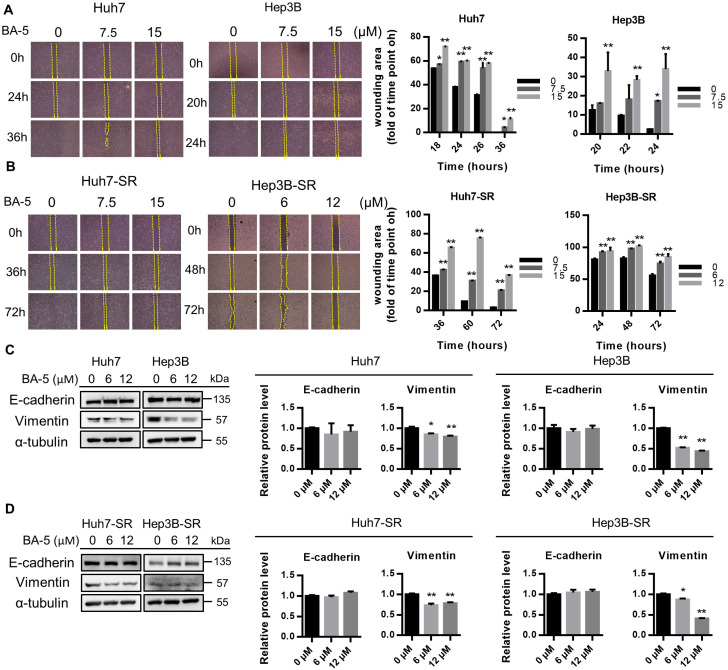
BA-5 treatment reduced the migration ability and Vimentin expression in HCC and HCC-SR cells. (**A**,**B**) HCC cells (2.8 × 10^5^/well Hep3B and 3.5 × 10^4^/well Huh7) and HCC-SR cells (2.8 × 10^5^/well Hep3B-SR and 1.75 × 10^5^/well Huh7-SR) were seeded in Ibidi culture inserts and treated with the indicated concentration of BA-5. The images were obtained at the indicated time point and wound areas were calculated by using Image J software and are shown in the column charts. (**C**,**D**) Hep3B, Huh7 (1.6 × 10^5^/well), Hep3B-SR and Huh7-SR (1.4 × 10^5^/well) cells were seeded in 6-well plates treated with 0, 6, and 12 µM BA-5 and incubated for 48 h. The protein expression levels of E-cadherin and Vimentin were evaluated by western blot. α-Tubulin served as a loading control. Protein quantification was performed by using Image J software. *, *p* < 0.05; **, *p* < 0.01 compared to the black bar.

Since EMT-related protein activation is associated with chemotherapy resistance in HCC, we further confirmed the effects of BA-5 on cell migration in HCC and HCC-SR cells. Cell migration was assessed by the wound healing assay, and the results showed that treatment with BA-5 inhibited cells migration and enhanced the wound area compared with the control in a time-dependent manner in both HCC and HCC-SR cells (Figure 5A,B). The protein expression of EMT-related markers was determined using Western blotting. As shown in Figure 5C,D, BA-5-treated cells significantly decreased the protein expression levels of Vimentin in a dose-dependent manner, while the expression of E-cadherin was not significantly changed. These data demonstrated that BA-5 inhibits HCC and HCC-SR cell migration by suppressing mesenchymal transition-associated proteins.

### 2.5. Genes and Biological Functions Affected by BA-5 Treatment in Both HCC and HCC-SR Cells

**Figure 6:**
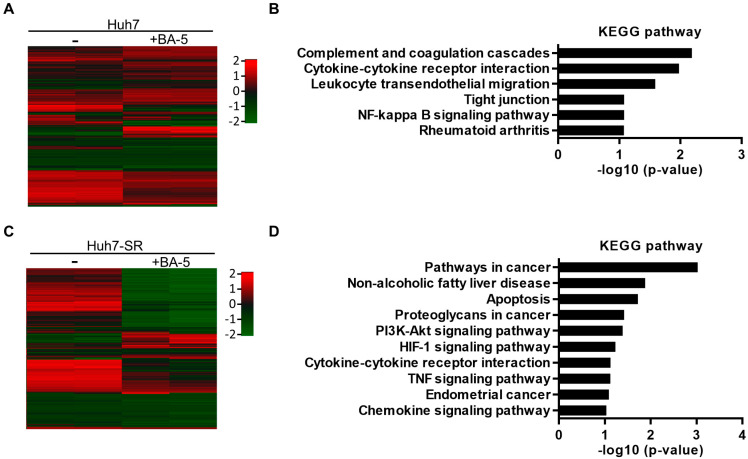
Gene expression profile of BA-5 treated Huh7 and Huh7-SR cells. (**A**,**C**) Heat map of differentially expressed genes in BA-5-treated Huh7 and Huh7-SR cells based on mRNA microarray analysis. A significant difference was observed in mRNA expression between the control and BA-5-treated cells. (**B**,**D**) Kyoto Encyclopedia of Genes and Genomes (KEGG) pathway enrichment analysis of up and downregulated genes between the control and BA-5-treated cells.

To analyze alterations in the gene expression profile after BA-5 treatment, Huh7 and Huh7-SR cells were treated with 15 μM of BA-5, and total RNA was extracted for microarray experiments. In the microarray analysis, a 2-fold increase or decrease in the signal intensity was considered a significant change in mRNA expression (Figure 6A,C). To reveal the pathways altered by BA-5 treatment, KEGG pathway enrichment analysis was performed. The results demonstrated that the altered genes in BA-5-treated Huh7 cells were enriched in pathways related to migration and the inflammatory response, including cytokine-cytokine receptor interaction, leukocyte transendothelial migration, tight junction, and NF-κB signaling pathway (Figure 6B). On the other hand, pathways related to cancer progression, including pathways in cancer, apoptosis, and the PI3K-AKT signaling pathway, were affected in BA-5-treated Huh7-SR cells (Figure 6D). We further analyzed the enriched up- and downregulated KEGG pathways of genes altered by BA-5 treatment in both Huh7 and Huh7-SR cells. As shown in Table 1, the HIF-1 signaling pathway was upregulated in BA-5-treated cells. In contrast, most pathways related to cancer progression were downregulated, including pathways in cancer, apoptosis, the PI3K-AKT signaling pathway, and regulation of the actin cytoskeleton. These results implied the possibility that BA-5 treatment inhibits cancer cell motility and growth.

### 2.6. BA-5 Suppressed Tumor Growth in an HCC Xenograft Mouse Model

**Figure 7:**
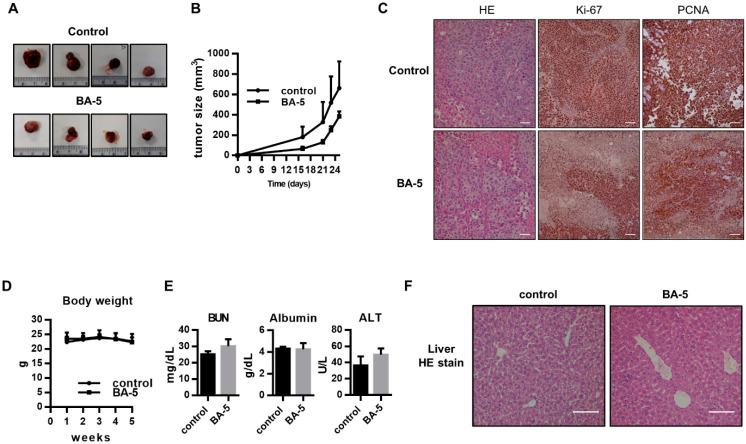
Antitumor effect of BA-5 in vivo. (**A**,**B**) Huh7 cells (1 × 10^6^) were injected subcutaneously into the right backs of male mice and randomly grouped into two groups, which were treated with or without BA-5 (4 mg/kg daily intraperitoneal injection). The images show tumors from each group. Tumors were measured twice weekly, and curves were calculated for each group. (**C**) Tumor sections were stained with H&E, Ki-67 and PCNA IHC. White bars equal to 0.1 mm. (**D**) The body weight change curve of mice measured weekly. (**E**) Serum BUN, ALT, and albumin levels detected by ELISA. (**F**) H&E-stained liver sections from mice treated with BA-5 and control groups. White bars equal to 0.1 mm.

To correlate the in vivo antitumor effects with the mechanism identified in vitro, an HCC xenograft mouse experiment was performed. We observed that BA-5-treated group tumor growth rate was lower than control group, and found tumor size of control group was smaller than that of control group (Figure 7A,B). However, there was no statistical difference between control group and BA-5-treated group. Histologically, the architecture of the tumor sections indeed resembled human HCC cell morphology, as demonstrated by H&E staining (Figure 7C). In addition, tumor sections in the BA-5-treated group had lower expression the proliferative biomarkers Ki-67 and PCNA than those in the control group (Figure 7C). These data implied that treatment with BA-5 inhibited tumor growth in the mouse model. To assess the side effects of BA-5 treatment, the body weight and serum levels of BUN, ALT, and albumin were analyzed. The results showed that there was no statistically significant difference between the BA-5-treated and BA-5-untreated groups (Figure 7D,E). In addition, liver sections from the BA-5 group showed regular arrangement compared with the control group (Figure 7F). These results suggested that BA-5 possessed antitumor ability and without any visible in vivo side effects.

### 2.7. The Combination of BA-5 with a Low Dose of Regorafenib Synergistically Inhibited HCC-SR Cell Viability

**Figure 8:**
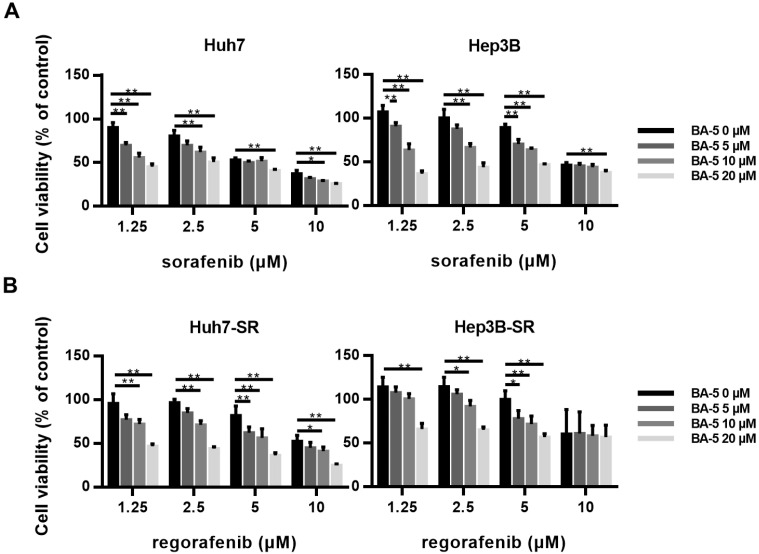
Treatment with combined BA-5 and sorafenib/regorafenib inhibited HCC and HCC-SR cell viability. (**A**) Huh7 and Hep3B cells (3 × 10^4^/well) were seeded in 96-well plates. After 48 h of treatment with BA-5 (0–20 µM) and sorafenib (1.25–10 µM), cell viability was detected by using an MTT assay. (**B**) Huh7-SR and Hep3B-SR cells (2.5 × 10^4^/well) were seeded in 96-well plates. After 48 h of treatment with BA-5 (0–20 µM) and regorafenib (1.25–10 µM), cell viability was detected by using an MTT assay. *, *p* < 0.05; **, *p* < 0.01.

Although sorafenib has been used for hepatocellular carcinoma treatment for more than a decade, long-term exposure to sorafenib often results in acquired resistance. Regorafenib has been approved by the FDA as second-line drug treatment for sorafenib-resistant patients [30]. However, the high cost of sorafenib and regorafenib has become a financial burden for HCC patients. Therefore, we further investigated whether combining BA-5 with sorafenib or regorafenib can synergistically inhibit HCC and HCC-SR cell proliferation, respectively. Treatment of parental HCC cells with sorafenib alone inhibited cell viability in a dose-dependent manner, while combining BA-5 with a lower dose of sorafenib (1.25, 2.5, 5 µM) significantly reduced cell growth (Figure 8A). As shown in Figure 8B, treatment of HCC-SR cells with regorafenib alone reduced cell viability in a dose-dependent manner, while combining BA-5 with a lower dose of regorafenib (1.25, 2.5, 5 µM) inhibited approximately 30–40% of cell growth, which was the same effect as regorafenib alone at 10 µM. The coefficient of drug interaction (CDI) was used to determine the type of interaction between the agents (Table 2). In Huh7 and Hep3B cells, 20 µM of compound BA-5 had a significantly synergistic effect with 1.25 µM sorafenib (CDI, 0.65 and 0.67, respectively). The similar synergistic result was also observed with the combined usage of BA-5 and sorafenib (2.5 µM) in Huh7 and Hep3B cells (CDI, 0.81 and 0.70, respectively). In Huh7-SR and Hep3B-SR cells, 20 µM of compound BA-5 had a synergistic effect with 1.25 µM regorafenib (CDI, 0.79 and 0.73, respectively) and 2.5 µM regorafenib (CDI, 0.75 and 0.74, respectively). These data imply that compound BA-5 was synergistic with a low dose of sorafenib or regorafenib in suppressing HCC and HCC-SR cells proliferation. These results showed that the cotreatment of BA-5 with a low dose of sorafenib or regorafenib may be a new strategy for the treatment of HCC and sorafenib resistant HCC.

## 3. Discussion

**Figure 9:**
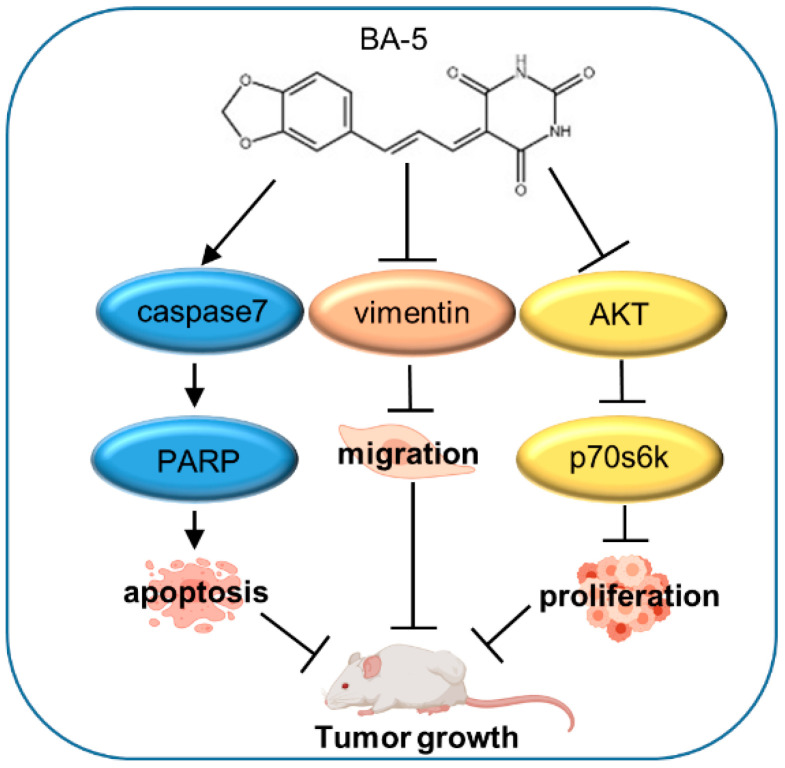
The proposed model demonstrating the role and mechanism of BA-5 in HCC and HCC-SR treatment.

Hepatocellular carcinoma accounts for 70–80% of liver cancer cases and is the third leading cause of cancer-related death [2]. Sorafenib is a standard first-line targeted drug approved for advanced HCC patients. However, the drug resistance hampers the use of sorafenib. The underlying mechanism of sorafenib resistance is complex, and its exact cause is still elusive [31]. Although sorafenib inhibits diverse kinases, a genetic investigation called attention to the compensatory activation of the AKT signaling pathway in the development of HCC, which might be a potential mechanism of acquired sorafenib resistance [32]. Reports have illustrated that sorafenib has limited benefit for 30% of patients [13]. In addition, erlotinib combined with sorafenib showed no significant difference in the survival rate compared to sorafenib alone in a clinical phase III trial [33]. Because sorafenib is expensive and has a low response rate, it is important to develop new drugs to treat HCC. In our study, we investigated the effect of a series of barbituric acid derivatives, among which BA-5 displayed more potent activity. In vitro experiments showed that BA-5 significantly inhibited HCC and HCC-SR cell viability, enhanced the apoptosis pathway, and suppressed migration activity. In the in vivo experiment, treatment with BA-5 reduced tumor growth in mice (Figure 9). 

Barbituric acid was first discovered by German chemist Adolph von Baeyer in 1864 and were the first active agents used clinically [16]. Barbiturates are also useful in widespread ways, including as sedative, anticonvulsant, and antiepileptic treatments [34]. Because barbituric acid derivatives have a variety of effects on sedation and have been used for their anesthetic, anticonvulsant, antibacterial, antifungal and antidiabetic activities [18,19,20,21,22], the pharmacology and chemistry of barbiturate have drawn great attention. In this study, we identified a novel barbituric acid derivative against HCC and sorafenib-resistant HCC. Cell proliferation plays a vital role in cancer development and progression [35]. Cancer cells show uncontrolled cell proliferation, promote cell proliferation without growth factor stimulation, escape apoptosis, and enhance migration to accelerate tumor progression [36]. Thus, antiproliferative activities provide targets for tumor therapy. Our results indicated that BA-5 inhibited cell viability in HCC and sorafenib-resistant HCC cells by blocking AKT signaling. The MAPK and AKT signaling pathways play a major role in cell proliferation, invasion, growth, and survival. These pathways are also involved in the pathogenesis and progression of a wide range of cancers, including HCC [37,38]. Recently, it has been demonstrated that phosphorylated AKT is related to hepatocellular recurrence and poor prognosis and is activated in 30–50% of HCC cases [39,40]. In addition, it has been found that increased AKT phosphorylation is correlated with acquired sorafenib resistance, while inhibition of AKT phosphorylation might reverse acquired sorafenib resistance [41,42,43]. Other studies also demonstrated that compounds based on using the barbituric acid structure as a framework model transferred to pyrimidine inhibitors or mTOR/PI3K inhibitors could reduce phospho-AKT expression [44,45]. In this study, we found that BA-5 consistently decreased phospho-AKT and phospho-p70s6k expression in HCC and HCC-SR cells.

Apoptosis is characterized by a series of morphologic changes, including cell shrinkage, nucleus fragmentation and chromatin condensation [46]. Caspase family proteins play a pivotal role in regulating the apoptosis pathway [47,48]. Furthermore, apoptosis-based strategies have been applied for cancer therapy [49]. Our data indicated that BA-5 enhanced cleaved caspase-7 activation and PARP cleavage. Some reports also showed that syntheses of skeletons began with barbituric acid and can induce cleaved PARP activation [26,45]. Epithelial-to-mesenchymal transition is a process in which cells lose cell junctions, change cell shape to fibroblast-like morphology, and acquire mesenchymal characteristics such as migration and invasion ability [50]. EMT is associated with the development of tumor invasion, dissemination, and metastasis [51,52]. Moreover, it has been found that EMT contribute to drug resistance in various cancers, including HCC [53]. Our results demonstrated that BA-5 inhibited the cell migration ability and reduced the protein expression level of Vimentin in both HCC and HCC-SR cells.

In Figure 2B, the result indicated that 20 uM BA-5 significantly inhibited cell viability in all cells. Morphology of attached cells was irregular shape and formed a lot of dead cells when cells were seeding in 6-well plate with 20 uM BA-5 for 48h. Therefore, we considered that 20 uM BA-5 wasn’t an appropriate concentration for follow-up functional mechanism. We choose different concentration 0, 6, 12 uM BA-5 treated cells for 48 h, and cell morphology was well and didn’t generate exceed dead cells. On the other hand, in gene expression experiment, cells were treated with BA-5 for 24 h to exact RNA. Considering the period treatment of BA-5 for RNA was only 24 h, we determined to increase the concentration of BA-5 up to 15 uM for gene analysis. 

Regorafenib as a second-line therapy approved in 2017 by the FDA for advanced HCC patients who are intolerant to sorafenib treatment. Regorafenib is a collection of receptor tyrosine kinase inhibitors, including PDGFR-β, FGFR-1, and VEGF1-3, and it is potentially stronger than sorafenib [30]. It has been reported that regorafenib reversed sorafenib resistance caused by HIF-induced EMT [54]. However, regorafenib also encountered drug resistance in patients with advanced HCC [55]. Our study showed that BA-5 not only inhibited cell viability when used alone but also had a synergistic effect with a lower dose of regorafenib, which decreased the viability of HCC-SR cells. In addition, the xenograft mouse model indicated that BA-5 suppressed tumor growth and did not produce any obvious negative consequences, such as liver injury and body weight loss. Taken together, our study illustrated that a new barbituric acid derivative, BA-5, inhibited tumor growth in a mouse model, and the underlying mechanism may be its regulatory function in inhibiting the AKT signaling pathway, lowering EMT occurrence and stimulating apoptosis activity. Therefore, these results implied that BA-5 is of prospective value for liver cancer therapy.

## 4. Materials and Methods 

### 4.1. Cell Culture

HepG2, Huh7, SK-Hep1 [56], and Hep3B (purchased from BCRC, Hsinchu, Taiwan, No. 60434) cells were grown in Dulbecco’s modified Eagle’s medium (DMEM; Gibco BRL, Grand Island, NY, USA) with 10% heat-inactivated fetal bovine serum (FBS, HyClone, Logan, UT, USA) at 37 °C in a humidified atmosphere containing 5% CO_2_. Sorafenib-resistant Huh7 (Huh7-SR) and sorafenib-resistant Hep3B (Hep3B-SR) cells were cultured from parental cells under increasing concentrations of sorafenib [57]. 

### 4.2. Drugs Treatment and the Coefficient of Drug Interaction

Compounds were dissolved in 100% dimethyl sulfoxide (DMSO; Sigma, St. Louis, MO, USA) and used at the concentrations indicated. Sorafenib Tosylate (purity > 98%, purchased from ApexBio (Houston, TX, USA)) and regorafenib (Toronto Research Chemicals, Toronto, Canada) were dissolved in 100% DMSO and used at the concentrations indicated. The coefficient of drug interaction (CDI) was used to analyze effects of drug combinations [58,59]. CDI is calculated as follows: CDI = AB/(A × B). According to the absorbance of each group, AB is the ratio of the combination groups to control group, where A or B is the ratio of the single agent group to the control group. Thus, CDI values less than, equal to, or greater than 1 indicates that the drugs are synergistic, additive, or antagonistic, respectively. CDI less than 0.7 indicates a significantly synergistic effect.

### 4.3. MTT Cell Viability Assay

HCC (3 × 10^4^/well) and HCC-SR (2.5 × 10^4^/well) cells were seeded into 96-well plates and cultured overnight. The culture medium was replaced with 20 μM BA-4a to BA-9 and incubated for 72 h. In the BA-5 alone treatment, the culture medium was replaced with BA-5 at the indicated concentration and incubated for 72 h. In the cotreatment experiment, the culture medium was replaced with BA-5 (0–20 μM) and regorafenib (1.25–10 μM) and incubated for 48 h. The culture medium was removed, and 50 μL of 1 × thiazolyl blue tetrazolium bromide (MTT reagent, Sigma-Aldrich, St. Louis, MO, USA) was added and incubated for 2.5 h at 37 °C in a 5% CO_2_ incubator. After incubation, the cells were treated with dimethy sulfoxide (Scharlab Chemie, Barcelona, Spain) for 10 min. Absorbance was detected at 570 nm. Untreated cells served as controls. The cell viability was calculated according to the formula: experimental OD value/control OD value × 100%.

### 4.4. Wound Healing Migration Assay

A culture insert from Ibidi (Martinsried, Germany) was used for the wound healing assays. HCC (2.8 × 10^5^/well of Hep3B and 3.5 × 10^4^/well of Huh7) and HCC-SR (2.8 × 10^5^/well of Hep3B-SR and 1.75 × 10^5^/well of Huh7-SR) cells were seeded in the culture insert to form a cell-free gap of approximately 500 μm. The culture insert and culture medium were removed and treated with different concentrations of BA-5 for the indicated hours. The gap area images were acquired and quantified (Image J, National Institutes of Health, Bethesda, MD) at the identified time point.

### 4.5. Western Blot Analysis

Total protein was extracted from cells lysed with cell lysis reagent (Sigma) containing protease inhibitors and phosphatase inhibitors. The protein concentrations of the cell lysates were measured using protein assay dye (Bio-Rad Laboratories, CA) at 595 nm. The amount of protein in all samples was normalized to 20 μg, separated by sodium dodecyl sulfate-polyacrylamide gel electrophoresis, transferred to polyvinylidene difluoride membranes and subsequently incubated with 5% milk with phosphate buffered saline for blocking. The membrane was incubated with primary antibody at 4 °C overnight. After incubation with peroxidase-conjugated secondary antibody for 1 h at room temperature at a concentration of 1:5000. The following antibodies used in this study were purchased from Cell Signaling (Beverly, MA, USA): phospho-, total-AKT, p70s6k, ERK, JNK, p38, caspase-3, caspase-7, caspase-8, PARP, Vimentin, and E-cadherin. The immunoreaction signals were normalized to those of α-tubulin (Sigma-Aldrich, St. Louis, MO, USA). The bands were visualized using an ECL detection reagent (Millipore Corporation, Billerica, MA, USA) and detected with an image acquisition system.

### 4.6. Gene Expression Profiling

The mRNA profiles were analyzed using Human OneArray Plus (Phalanx Biotech, Hsinchu, Taiwan). Total RNA was extracted from Huh7 and Huh7-SR cells. Whole-genome gene expression was measured in these samples using OneArray Plus chips (Phalanx Biotech Group). Hierarchical clustering was performed using Cluster 3.0 (http://bonsai.hgc.jp/~mdehoon/software/cluster/). The differential expression of genes listed in the hierarchical clustering map was defined by the ratio of the expression in BA-5 untreated cells to that in BA-5-treated cells as a log2 (fold change) of ≥ 2 (upregulation) or ≤ 0.5 (downregulation). The gene expression patterns in different pathways were analyzed using the KEGG pathway database (https://www.genome.jp/kegg/pathway.html).

### 4.7. Subcutaneous Xenograft Model

Male and female NOD.CB17-Prkdcscid/jNarl mice (5 weeks old) were purchased from Taiwan National Laboratory Animal Center. The protocol was reviewed and approved by the Institutional Animal Care and Use Committee of Taipei Medical University (LAC-2017-0347) in January 2018. Huh7 cells (1 × 10^6^) were subcutaneously inoculated into the right backs of male mice. Mice were randomly grouped into two groups (*n* = 5/group) after Huh7 cell injection, which were treated with or without 2 mg/kg BA-5 by intraperitoneal injection 5 times a week. Tumors were measured twice weekly by using a Vernier caliper to measure the length (L) and width (W) of the tumors. The tumor volume (TV) was calculated as follows: TV = (L × W^2^)/2. Body weight was recorded twice weekly. 

### 4.8. Immunohistochemistry (IHC) Staining and Blood Biochemical Parameters

The mouse tissues were fixed with 4% formaldehyde and dehydrated in graded ethanol and xylene. The tissues embedded in paraffin were sliced into 5 μm sections, which were incubated with anti-Ki-67 (1:100) and anti-PCNA (1:100) antibodies for 1.5 h (GenScript, Piscataway, NJ, USA). After washing, sections were reacted with a Universal LSAB2 kit (DakoCytomation Carpinteria, CA, USA). The images were taken using a light microscope. Serum alanine aminotransferase (ALT), albumin, and blood urea nitrogen (BUN) values were measured with a biochemical analyzer (VetTest™, IDEXX, USA).

### 4.9. Statistical Analysis

Student’s t test was used to evaluate the significance of the difference between experimental groups and the control group, between combination groups, and corresponding sole drug groups, where *p*-values of <0.05 were considered significant.

## Figures and Tables

**Table 1:** KEGG pathway enrichment analysis of BA-5 treated cells.

Description	Count	%	*p* Value
**Upregulation**			
HIF-1 signaling pathway	3	7.1	0.016
**Downregulation**			
Pathways in cancer	15	7.2	0.00037
TNF signaling pathway	7	3.3	0.0022
Cytokine-cytokine receptor interaction	10	4.8	0.0034
Apoptosis	5	2.4	0.0076
Rap1 signaling pathway	8	3.8	0.017
PI3K-Akt signaling pathway	10	4.8	0.03
Signaling pathways regulating pluripotency of stem cells	6	2.9	0.032
Central carbon metabolism in cancer	4	1.9	0.047
Regulation of actin cytoskeleton	7	3.3	0.049
p53 signaling pathway	4	1.9	0.052
Complement and coagulation cascades	4	1.9	0.056
Leukocyte transendothelial migration	5	2.4	0.057
Melanoma	4	1.9	0.06
Phenylalanine, tyrosine and tryptophan biosynthesis	2	1.0	0.062
Sphingolipid signaling pathway	5	2.4	0.065
Chemokine signaling pathway	6	2.9	0.086
FoxO signaling pathway	5	2.4	0.089

**Table 2:** CDI of the combination of BA-5 and sorafenib/regorafenib in parental and sorafenib-resistant liver cancer cells.

BA-5 (20 µM)					
Sorafenib (µM)	Huh7	Hep3B	Regorafenib (µM)	Hhh7-SR	Hep3B-SR
1.25	0.65	0.67	1.25	0.79	0.73
2.5	0.81	0.70	2.5	0.75	0.74
5	0.92	0.84	5	0.74	0.82
10	0.89	1.15	10	0.81	1.05
